# Synthesis of Ni_4.5_Fe_4.5_S_8_/Ni_3_S_2_ film on Ni_3_Fe alloy foam as an excellent electrocatalyst for the oxygen evolution reaction†

**DOI:** 10.1039/c9ra00724e

**Published:** 2019-04-02

**Authors:** Shili Qin, Jinlong Lei, Yun Xiong, Xiaohu Xu, Xinhua Geng, Jiahai Wang

**Affiliations:** Department of Chemistry and Chemical Engineering, Guangzhou Key Laboratory for Environmentally Functional Materials and Technology, Guangzhou University Guangzhou 510006 P. R. China ccgxinh1230@gzhu.edu.cn jiahaiwang@gzhu.edu.cn +86-18816801579; Wuhan Economic and Technological Development Zone, Wuhan HydraV Fuel Cell Tech. Co., Ltd Wuhan 430056 P. R. China; Key Laboratory of Spectral Measurement and Analysis of Shanxi Province, Shanxi Normal University Linfen 041004 P.R. China

## Abstract

Directly synthesizing bicomponent electrocatalysts in the nanostructured form from bulk alloy foam has many potential advantages: robust stability, synergistic effects and fast electron transfer. Here, Ni_4.5_Fe_4.5_S_8_/Ni_3_S_2_ film with micrometer thickness on bulk substrate was synthesized by a simple one-step hydrothermally assisted sulfurization of Ni_3_Fe alloy foam for the oxygen evolution reaction (OER) in basic media. Benefiting from the synergetic effect of the bicomponent, reduced interfacial resistance between electrocatalyst and metal substrate, and more exposed catalytic sites on the microstructured film, the as-prepared electrocatalyst (Ni_4.5_Fe_4.5_S_8_/Ni_3_S_2_‖Ni_3_Fe) behaves as a highly efficient and robust oxygen evolution electrode with felicitous current density in alkaline electrolytes (1 M KOH). It requires an overpotential of only 264 mV to drive 100 mA cm^−2^ with its catalytic activity being maintained for at least 20 h in 1 M KOH. In the near future, this kind of synthesis strategy can be easily extended to investigate many electrocatalysts derived from 3D alloyed foam with various ratios of the different components, opening new avenue for understanding the relationship between material properties and electrochemical performance.

## Abstract 2

Ni_4.5_Fe_4.5_S_8_/Ni_3_S_2_‖Ni_3_Fe composite materials show excellent OER electrocatalytic performance in alkaline solutions.

## Graphical abstract

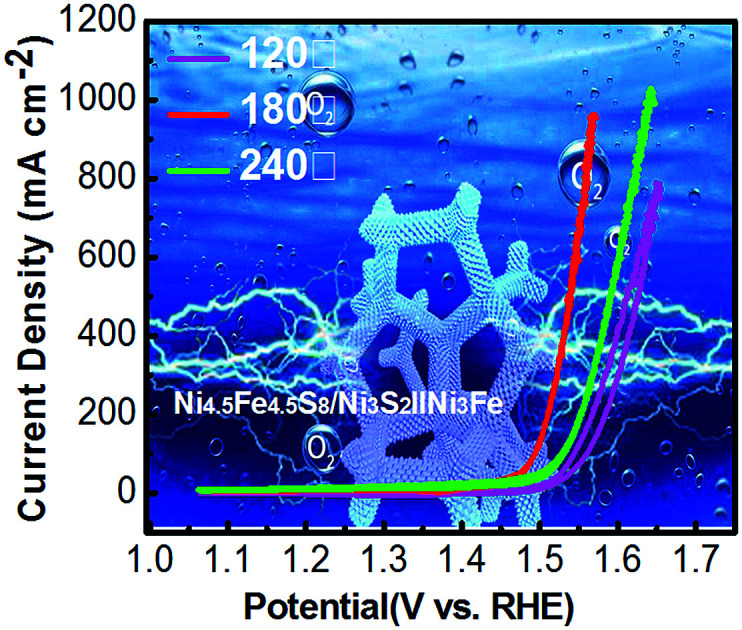

## 1. Introduction

The energy crisis and environmental issues are critical challenges, due to limitations of fossil fuels and population growth.^1–5^ In order to deal with these problems, research on new energy sources is necessary. New energy sources such as solar and wind energies are highly promising alternatives, but their use is limited by their intermittencies and geographically uneven distributions. Thus, suitable energy storage strategies are needed.^6–8^ Electrochemical water-splitting is one of the principle methods to garner hydrogen fuel as a clean and abundant energy resource with its high energy storage density as well as zero carbon emission.^9^ Currently, the real voltage for water splitting is much higher than the theoretical value (1.23 V) because of the intrinsic activation barrier of the catalytic centres, interfacial resistance and other voltage losses in the electric circuit. The full water splitting involves two half-reactions, whose overpotentials are further complicated by the high intrinsic activation barriers inherent to the two half reactions involved in water splitting, *i.e.*, the hydrogen evolution reaction (HER) and the oxygen evolution reaction (OER).^10–12^ In particular, the overall rate is heavily dependent on the OER catalyst, due to the sluggish kinetics and complex reaction pathway of the OER.^10,13,14^ Up to now, noble metal oxides (RuO_2_ and IrO_2_) are considered as benchmarked catalysts, but their high cost and scarcity severely impede their wide applications.^2,10,15–19^

Recently, much effort has focused on inexpensive and earth-abundant elements-based materials as viable alternatives for OER, such as transition-metal sulfides, selenides, oxides, nitrides and (oxy)-hydroxides.^13,15,20–29^ It has been demonstrated that transitional-metal chalcogenides exhibit enhanced conductivity and the electroactive performance can be further ameliorated by doping other elements, deriving hydro(oxide) nanoshell and forming nanostructures. Doping single phase with other elements can induce the electron transfer between each element, leading to the further modulation of the adsorption of hydrogen(oxygen) molecules on the active sites^22,30–32^ and of the kinetic energy barrier of hydrogen(oxygen) evolution pathway. Previously, Xu *et al.* synthesizes Fe-doped Ni_3_S_2_ from NiFe foil, which requires overpotential of 282 mV to achieve the current density of 10 mA cm^−2^.^33^ The second approach for enhancing the performance of single active site can be realized by constructing new interfaces with two components, which have been demonstrated by several studies.^34–36^ The second component can adjust the electron density around the active sites in the first layer *via* the new formed interface. In some cases, the new interface allows the two components to act synergistically: one component allows H_2_O(OH^−^) adsorption and the other component facilitates the O_2_(H_2_) release.^34,37–39^ It has reported in several cases that some chalcogenide-based electrocatalysts intend to transform into metal (hydro)oxides, which is verified to be active catalytic centre on the surface.^40,41^ Controlled formation of electroactive nanoshell on the surface has the unique advantage, retaining the electroactive performance and the excellent bulk conductivity simultaneously. For majorities of the chalcogenide-based electrocatalysts developed recently, designing materials in nanostructured form further enhances the overall performance^41,42^ by exposing more active sites in the solid–electrolyte interface.

Direct sulfurization of 3D metal foam has been proved to be an effective method to synthesize the catalysts which is seamlessly integrated with bulk conductive materials. Notably, X. Zou *et al.* have demonstrated that, using NF as support, Ni_3_S_2_ can be formed *via* one-step hydrothermal sulfides method, the exposed (210) facet leading to an excellent catalytic activity of Ni_3_S_2_ film.^43^ Using NF as support, Sun's group has synthesized Fe-doped Ni_3_S_2_ particle, benefited from the doping effects, the as-prepared material shown excellent performance and stability.^31^ However less reports have presented one-step chemically derivation of 3D alloy foam. After chemically derivation of alloy foam, it is highly possible to form new interface between two components.^44^ Furthermore, the excellent conductivity is still retained because of the integration with bulk alloy form. Most importantly, adjusting the ratio between each element in the alloy foam has the potential to form totally different scenario. Previous works normally synthesize two chalcogenide-based components with newly formed interface by electrodepositing single component precursor or by hydrothermally preparing single component precursor.^37,45,46^ The single component precursor contains at least two metal elements in the ion form. Liu *et al.* successfully prepared NiFeS electrocatalyst with further sulfurization process of NiFe hydroxides precursor, which showed superior OER performance.^42^

Herein, we report, for the first time, the synthesis of stable and high-performance Ni_4.5_Fe_4.5_S_8_/Ni_3_S_2_ hybrid materials supported on alloy foam (Ni_3_Fe), dubbed Ni_4.5_Fe_4.5_S_8_/Ni_3_S_2_‖Ni_3_Fe. One step hydrothermal method was used to prepare the Ni_4.5_Fe_4.5_S_8_/Ni_3_S_2_ composite, which is easy and efficient for upscale production without adding external metal ions. Nickel-iron foam was selected as a scaffold to form 3D conductive networks and promote electronic conductivity, exposing a large surface area of active sites. In addition, the synergistic interaction between Ni_4.5_Fe_4.5_S_8_ and Ni_3_S_2_ can improve overall properties of the material. Therefore, in the alkaline media, the prepared Ni_4.5_Fe_4.5_S_8_/Ni_3_S_2_‖Ni_3_Fe electrode showed superior electrocatalytic activity toward OER, which showed a small onset potential of only 1.38 V (RHE) and a low overpotential of only 166 mV to reach a current density of 10 mA cm^−2^. Additionally, Ni_4.5_Fe_4.5_S_8_/Ni_3_S_2_‖Ni_3_Fe also showed a small Tafel slope of 63.31 mV dec^−1^ and excellent stability.

## 2. Experimental details

### 2.1. Materials

Hydrochloric acid (HCl), potassium hydroxide (KOH), thiourea, acetone and ethanol were acquired from Sinopharm Chemical Reagent Co., Ltd. RuO_2_ and Nafion (5 wt%) was purchased from Shanghai Macklin Biochemical Co., Ltd. Ni_3_Fe alloy foam, Ni foam and Fe foam were received from Kun Shan Kunag Xun Electronics Co., Ltd., China. All chemicals were of analytical grade and used as received without further purification. The water used throughout all experiments was purified through a Millipore system.

### 2.2. Synthesis of Ni_3_S_2_‖NF, FeS_2_‖Fe and Ni_4.5_Fe_4.5_S_8_/Ni_3_S_2_‖Ni_3_Fe

Firstly, Ni_3_Fe alloy foam (1.4 × 3.3 cm^2^) was washed with acetone by sonication for 10 minutes, then the Ni_3_Fe alloy foam was treated with 3 M HCl by sonication for 10 minutes, finally, the Ni_3_Fe alloy foam was washed with water three times to get clean Ni_3_Fe samples. To synthesize Ni_4.5_Fe_4.5_S_8_/Ni_3_S_2_‖Ni_3_Fe electrode, 0.8 g of thiourea was dissolved in 35 mL of deionized water under magnetic stirring for 15 minutes. Then the freshly prepared thiourea solution was transferred to a Teflon-lined stainless steel autoclave (50 mL) containing a piece of pretreated Ni_3_Fe alloy foam into the solution. The autoclave was sealed and then heated at 180 °C for 16 h. After the autoclave cooled down to room temperature naturally, the obtained Ni_4.5_Fe_4.5_S_8_/Ni_3_S_2_‖Ni_3_Fe was washed with de-ionized water several times and then dried in vacuum. For comparison, Ni_3_S_2_‖Ni and FeS_2_‖Fe electrodes were also fabricated using the same procedure except that Ni foam or Fe foam was used as current collector and metal source.

### 2.3. Materials characterization

The crystal structure of the as-prepared Ni_3_Fe, and Ni_4.5_Fe_4.5_S_8_/Ni_3_S_2_‖Ni_3_Fe samples have been measured by X-ray powder diffraction (XRD, Bruker D8 ADVANCE, Cu KR). The morphology and phase information of Ni_4.5_Fe_4.5_S_8_/Ni_3_S_2_‖Ni_3_Fe samples were examined by transmission electron microscopy (JEM-2100), field-emission scanning electron microscopy (SEM, Hitachi, S-4800) and corresponding selected area electron diffraction (SU8010). XPS was performed on a Thermo Scientific Escalab 250Xi spectrometer using an Al Kα photon-source.

### 2.4. Electrochemical characterization

All of the electrochemical measurements were carried out with a CHI660E electrochemical workstation in 1.0 M KOH electrolyte solution. The as-prepared Ni_4.5_Fe_4.5_S_8_/Ni_3_S_2_‖Ni_3_Fe were used directly as the working electrode without further treatments. Cyclic Voltammetry (CV) measurements were performed at the potential ranging from 0.0 to 1.0 V (*vs.* SCE) at a scan rate of 5 mV s^−1^. Electrochemical impedance spectroscopy (EIS) determination was conducted in the frequency range from 1.0 × 10^5^ Hz to 1.0 Hz. The geometric surface area of the electrode is 0.2 cm^2^. All the measured current densities were obtained by dividing the current with the geometric surface area. The electrochemical characterizations were carried in a standard three-electrode system by using a graphite bulk counter electrode and a Hg/Hg_2_Cl_2_ (sat. KCl) reference electrode. The potentials reported here were calibrated with respect to the reversible hydrogen electrode (RHE): *E*(RHE) = *E*(Hg/Hg_2_Cl_2_) + 0.0591 × pH + 0.2415 − 0.000761(*T* − 298.15).

## 3. Results and discussions

### 3.1. Morphology and composition characterization

**Scheme 1:**
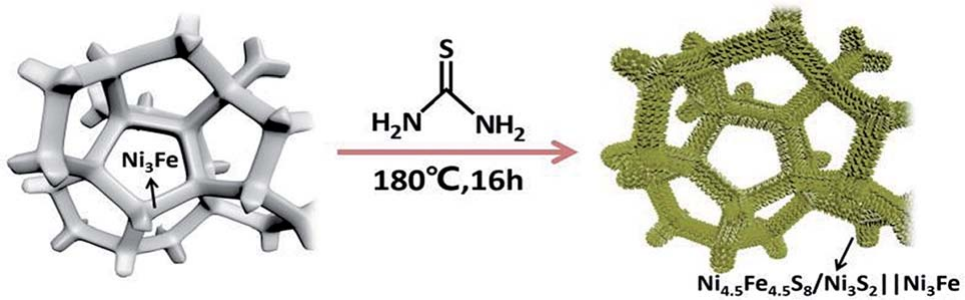
Schematic illustration of preparation of Ni_4.5_Fe_4.5_S_8_/Ni_3_S_2_ composite on Ni_3_Fe foam.

**Fig. 1:**
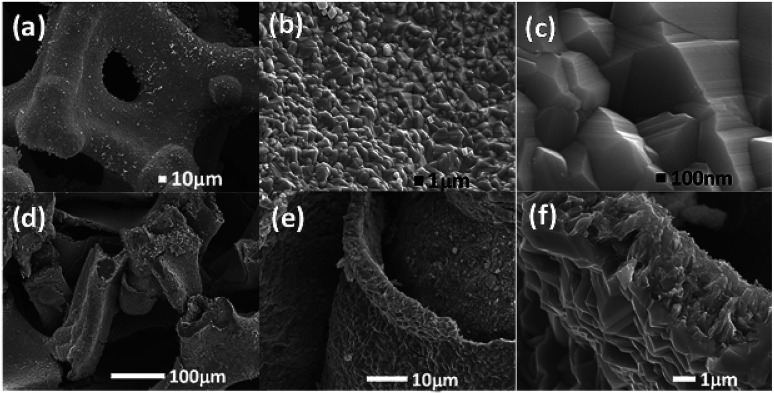
(a–c) SEM images of the Ni_4.5_Fe_4.5_S_8_/Ni_3_S_2_‖Ni_3_Fe mountainous terrain on Ni_3_Fe alloy foam. (d–f) Cross section of Ni_4.5_Fe_4.5_S_8_/Ni_3_S_2_‖Ni_3_Fe composite material.

**Fig. 2:**
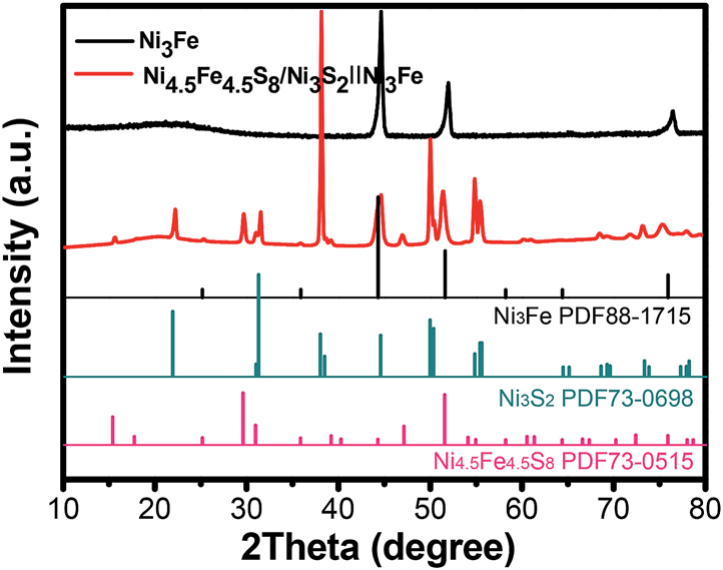
XRD pattern of the as-synthesized Ni_4.5_Fe_4.5_S_8_/Ni_3_S_2_‖Ni_3_Fe.

**Fig. 3:**
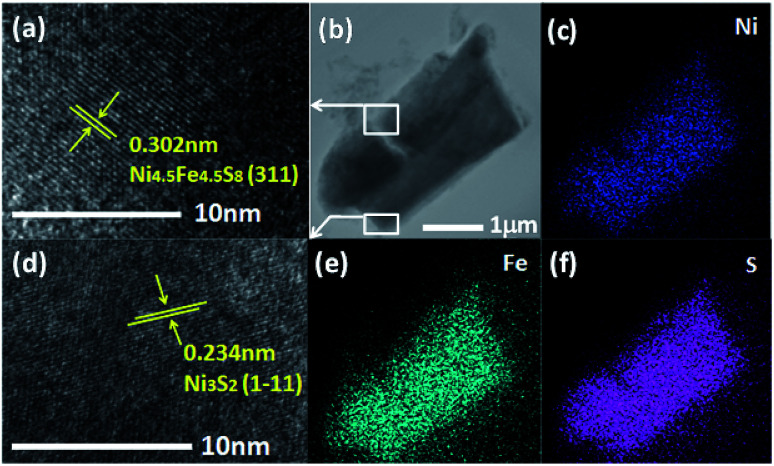
(a and d) High resolution TEM image of Ni_4.5_Fe_4.5_S_8_/Ni_3_S_2_‖Ni_3_Fe. (b) Low resolution TEM image resolution of Ni_4.5_Fe_4.5_S_8_/Ni_3_S_2_‖Ni_3_Fe. (c, e and f) Elements mapping (Ni, Fe, S) in Ni_4.5_Fe_4.5_S_8_/Ni_3_S_2_‖Ni_3_Fe, respectively.

As shown in Scheme 1, one-step synthesis of Ni_4.5_Fe_4.5_S_8_/Ni_3_S_2_ on Ni_3_Fe foam without addition of extra metal ions is quite straightforward. After hydrothermal sulfurization of alloy foam, mountainous terrain (Fig. 1) on macroporous foam is formed, which is totally different from the bare smooth surface of Ni_3_Fe foam (Fig. S1†). The sample fragment scraped down from the alloy foam shows that the composite has clear edges in the low-resolution TEM image (Fig. 3b). In combination with the XRD pattern in Fig. 2, it can reason that the mountainous terrain is mainly composed of Ni_4.5_Fe_4.5_S_8_ and Ni_3_S_2_. More detailed information can be gleaned from the X-ray diffraction (XRD) patterns (Fig. 2). As for XRD pattern measured from Ni_3_Fe foam, the peaks at 44.2°, 51.5°, 75.8° are corresponding to (111), (200), (220) planes of Ni_3_Fe (JCPDS 88-1715). After sulfurization of alloy foam, many new peaks have appeared. The peaks at 21.8°, 31.2°, 37.8°, 44.4°, 49.8° can be ascribed to (100), (110), (111), (200), (210) planes of Ni_3_S_2_ (JCPDS 73-0698) and the peaks at 15.3°, 29.5°, 30.8°, 47.0°, 51.5° correspond well with (111), (311), (222), (511), (440) planes of Ni_4.5_Fe_4.5_S_8_ (JCPDS 73-0515). Therefore, it is confirmed that the formed product has two components (Ni_4.5_Fe_4.5_S_8_ and Ni_3_S_2_). No other impurities in the samples were revealed in XRD measurements.

In order to further confirm the crystal phase and chemical composition of the as-prepared catalyst. A scanning transmission electron microscopy (STEM) was utilized for measurements. As shown in Fig. 3a and d, lattice fringes with interplanar distance of 0.3 nm were clearly revealed in the high-resolution image, which corresponds to the (311) plane of Ni_4.5_Fe_4.5_S_8_, the distance of 0.234 nm can be ascribed to the (1−11) plane of the Ni_3_S_2_ phase, respectively. It indicates that the Ni_4.5_Fe_4.5_S_8_/Ni_3_S_2_ phase was synthesized successfully in accordance with the XRD. Elemental mapping images in Fig. 3c, e and f and EDS pattern (Fig. S4†) illustrate the presence of Ni, Fe and S, further proofing the existence of Ni_4.5_Fe_4.5_S_8_/Ni_3_S_2._

### 3.2. Surface chemistry

**Fig. 4:**
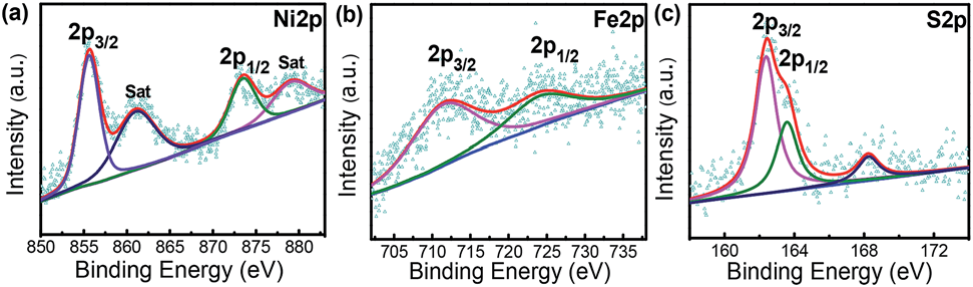
XPS spectra for (a) Ni 2p. (b) Fe 2p. (c) S 2p of Ni_4.5_Fe_4.5_S_8_/Ni_3_S_2_‖Ni_3_Fe.

X-ray photoelectron spectroscopy (XPS) analysis is an effective approach to analyse the surface element valence and chemical bonding environments in the composite. A survey of XPS spectrum confirms the Ni_4.5_Fe_4.5_S_8_/Ni_3_S_2_‖Ni_3_Fe sample contains Fe, Ni, and S atoms. The Ni 2p XPS peaks located at 855.62 eV and 873.47 eV can be assigned to Ni^2+^ 2p_3/2_ and 2p_1/2_ binding energies, respectively (Fig. 4a). These values are consistent with those for Ni^2+^ in Ni_4.5_Fe_4.5_S_8_/Ni_3_S_2_.^33,47–49^ The peaks corresponding to the binding energy of Fe 2p_1/2_ and Fe 2p_3/2_ at 724.04 and 711.24 eV demonstrate the trivalent state of Fe in the composite (Fig. 4b).^31,42,50,51^ The peaks of S 2p at 162.39 and 163.56 eV were attributed to negative bivalent S 2p_3/2_ and S 2p_1/2_, respectively (Fig. 4c), indicating the bivalent state of S,^33,52^ The peak at 168.25 eV is well matched to the highly oxidized state S^4+^ at the edge of Ni_4.5_Fe_4.5_S_8_/Ni_3_S_2_.^53^ The above results confidently confirm the successful synthesis of Ni_4.5_Fe_4.5_S_8_/Ni_3_S_2_.

### 3.3. Electrocatalytic activity

**Fig. 5:**
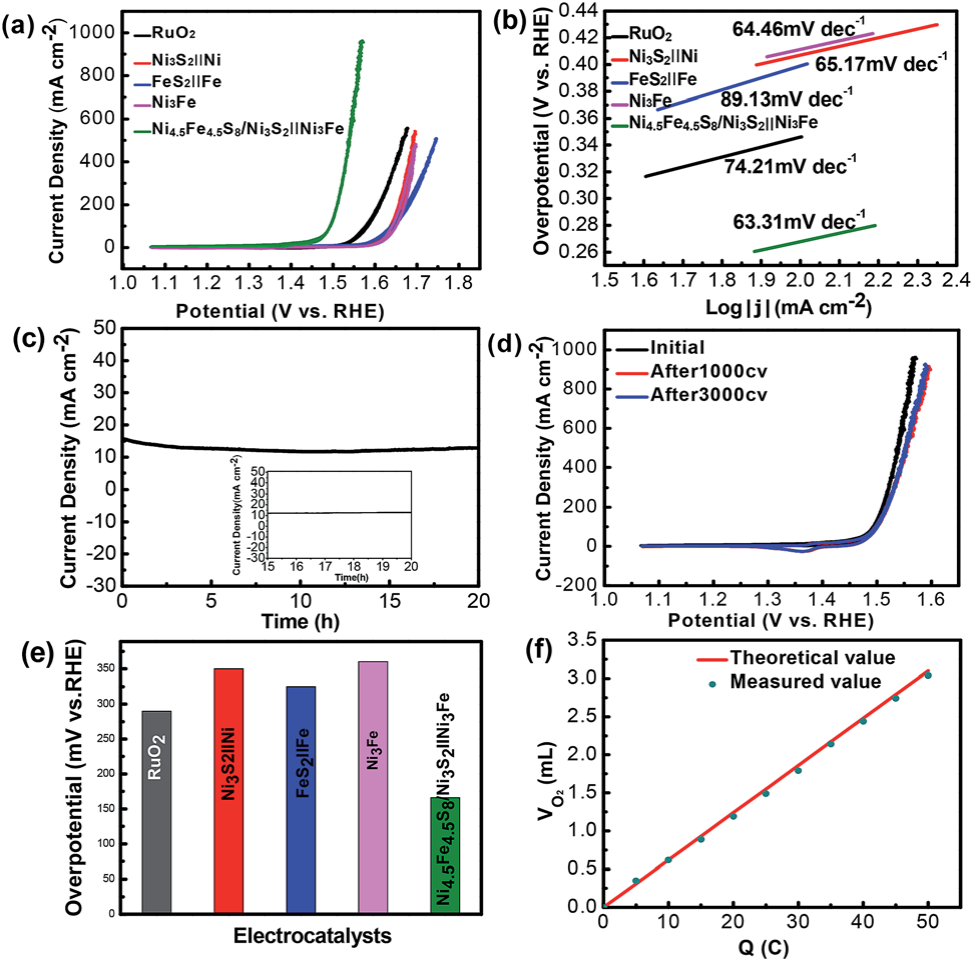
(a) CV polarization curves for OER. (b) Tafel plots with the corresponding Tafel slopes for the OER process. (c) Chronoamperometric durability test at a constant overpotential of 215 mV. (d) The CV curves before and after the durability test. (e) Overpotentials at current density of 10 mA cm^−2^. (f) Quantitative O_2_ measurement *via* water displacement.

For each sample measured in three-electrode system, Cyclic Voltammetry (CV) were measured to assess the electrocatalytic performance in 1 M KOH solution (Fig. 5a). For comparison, several control samples such as bare Ni_3_Fe foam, Ni_3_S_2_‖Ni, FeS_2_‖Fe and RuO_2_ (loading amount: 6 mg cm^−2^) were tested. Fig. 4a shows the CV curves for OER of the tested samples at a scan rate of 5 mV s^−1^. Remarkably, the samples (Ni_4.5_Fe_4.5_S_8_/Ni_3_S_2_‖Ni_3_Fe) prepared at 180 °C show the best performance (Fig. S2†) and the bare Ni_3_Fe foam only manifests negligible activity at overpotential of 300 mV. Ni_4.5_Fe_4.5_S_8_/Ni_3_S_2_‖Ni_3_Fe possesses the ability to deliver current density of 10 and 100 mA cm^−2^ at overpotential of 166 and 264 mV, respectively, outperforming the above-mentioned control samples. FeS_2_‖Fe, Ni_3_S_2_‖Ni and RuO_2_ require overpotential of 330, 350 and 360 mV to achieved current density of 10 mA cm^−2^ (Fig. 5e). These results confirm that the electrocatalyst prepared in this study has enhanced activity after sulfurization and is even superior to some noble-metal-free electrocatalysts. It is proposed that direct seamless integration of electrocatalyst with conductive Ni_3_Fe alloy substrate plays an important role for the improved performance of OER. The simultaneous presence of Fe and Ni allows the opportunity to synthesize bicomponent electrocatalyst with potential synergistic effect. After OER tests, the Ni in Ni_4.5_Fe_4.5_S_8_/Ni_3_S_2_‖Ni_3_Fe was oxidized to metal oxides by electro-chemical oxidation in alkaline solution, the valence of Ni 2p_3/2_ and Ni 2p_1/2_ also shifted to higher state 856.79 and 874.53 eV and two satellite peaks at 862.65 and 880.66 eV, the energy separation between Ni 2p_3/2_ and Ni 2p_1/2_ is ≈17.7 eV, which indicated the existence of NiO phase by XPS spectra (Fig. S6a†).^35,51,54^ Furthermore, the valence of Fe also shifted to higher state 712.40 eV and 725.30 eV with new satellites 718.31 eV and 731.49 eV related with Fe_2_O_3_ phase (Fig. S6b†)_._^42,55^ Deconvolution of oxygen peak revealed one new peak as compared to OER test, indicating formation of oxygen–metal bond (Fig. S7†).^56–59^ From these analyses, it can reason that new metal-hydro(oxide) phase was produced after OER test. Furthermore, the new peak at 167.80 eV in XPS of S 2p verified the oxidation of sulfur to SO_4_^2−^ (Fig. S6c†).^47,60^ A slight degradation of catalytic performance after 1000 cycles of CV test can be ascribed to the oxidation of catalytic surface (Fig. 5d).

Tafel slope as a quality descriptor derived from the CV curves is used to investigate the OER catalytic kinetic performance of the samples. As shown in (Fig. 5b), the electrocatalyst (Ni_4.5_Fe_4.5_S_8_/Ni_3_S_2_‖Ni_3_Fe) shows fastest kinetics at the onset potential, demonstrated by the Tafel slope (63.31 mV dec^−1^). The Tafel slope value of Ni_4.5_Fe_4.5_S_8_/Ni_3_S_2_‖Ni_3_Fe is smaller than those of FeS_2_‖Fe (89.13 mV dec^−1^), Ni_3_S_2_‖Ni (65.17 mV dec^−1^), RuO_2_ (74.21 mV dec^−1^) and Ni_3_Fe alloy (64.46 mV dec^−1^). The long-term durability (Fig. 5c, d and S3†) of Ni_4.5_Fe_4.5_S_8_/Ni_3_S_2_‖Ni_3_Fe for OER was assessed by applying constant overpotential of 215 mV for 20 h. After 20 h of constant overpotential test, the current density of 15 mA cm^−2^ does not change significantly, the CV curves after 1000 CV cycles has only slight shifted as compared to that before cyclic scanning, manifesting its outstanding stability for OER. The XRD characterization after stability test shows the same pattern as that before electrochemical measurements (Fig. S5†). The same mountainous terrain was retained with appearance of much rougher surface possibly because of surface oxidation. XPS also reconfirmed the same trend of surface oxidation. Nevertheless, the recorded CV curve after 3000 cycles of CV almost overlapped with that after 1000 cycles. Undoubtedly, this catalyst can stabilize after formation of metal-hydro(oxide) shell without further degradation. The existence of XPS peaks of sulfur after OER stability test is an excellent evidence for retainment of Ni_4.5_Fe_4.5_S_8_ and Ni_3_S_2_.

**Fig. 6:**
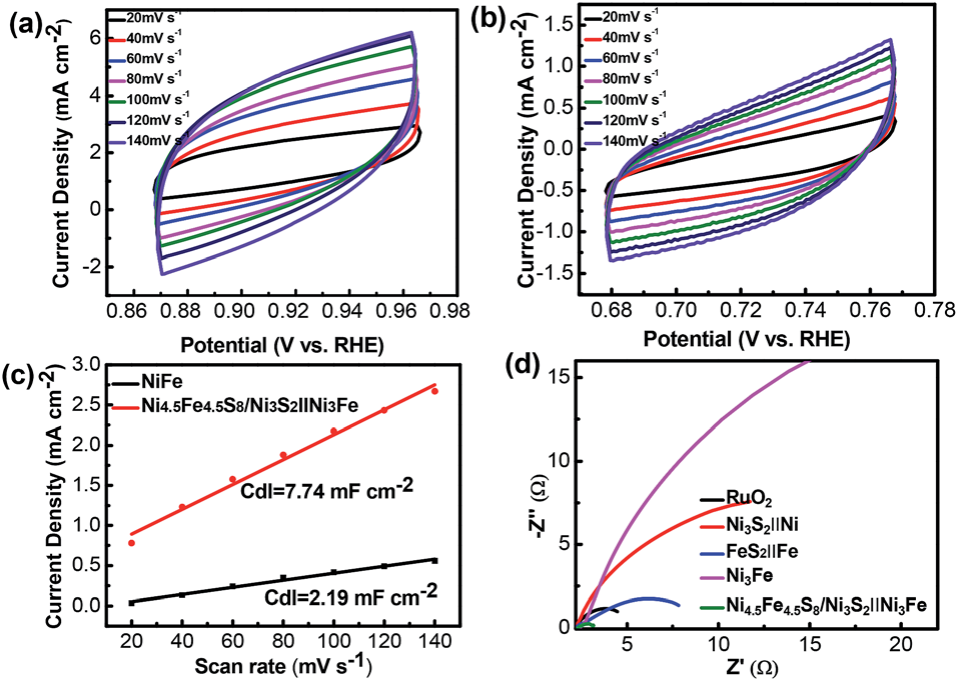
(a and b) CV curves of the Ni_4.5_Fe_4.5_S_8_/Ni_3_S_2_‖Ni_3_Fe and Ni_3_Fe electrodes measured in 1.0 M KOH in the non-faradaic region with different scan rates from 20 to 140 mV s^−1^. (c) Current density differences (Δ*J*/2) plotted against scan rates of Ni_4.5_Fe_4.5_S_8_/Ni_3_S_2_‖Ni_3_Fe and Ni_3_Fe. (d) Nyquist plots of different catalysts in 1.0 M KOH.

The electrochemically active surface area (ECSA) of electroactive materials can be obtained by measuring the electrochemical double-layer capacitance (*C*_dl_), given the fact that *C*_dl_ is proportional to the ECSA. The *C*_dl_ was obtained through cyclic voltammetric scans at different rates (20 to 140 mV s^−1^) and in the non-faradaic potential range. *C*_dl_ increases from 2.19 mF dec^−1^ for Ni_3_Fe alloy foam to 7.74 mF dec^−1^ for Ni_4.5_Fe_4.5_S_8_/Ni_3_S_2_‖Ni_3_Fe, indicating that Ni_4.5_Fe_4.5_S_8_ and Ni_3_S_2_ improved the electrochemical active surface area, thus contributing to the improved electrocatalytic activity (Fig. 6a–c). Electrochemical impedance spectroscopy (EIS) was further used to investigate the charge transfer kinetics at the catalyst–electrolyte interface during the OER.

Apparently, it is shown in Fig. 6d that the values of resistance charge transfer (*R*_ct_) for the five samples are arranged in the order of Ni_4.5_Fe_4.5_S_8_/Ni_3_S_2_‖Ni_3_Fe < RuO_2_ < FeS_2_‖Fe < Ni_3_S_2_‖Ni < Ni_3_Fe, the smaller *R*_ct_ value reveals faster charge transport of Ni_4.5_Fe_4.5_S_8_/Ni_3_S_2_‖Ni_3_Fe during the OER catalytic process, which is beneficial to the generation of O_2_. Although the *C*_dl_ is a relatively small value, the fast electron transfer and high turnover frequency of single active site definitely offset this disadvantage. Moreover, the final activity of Ni_4.5_Fe_4.5_S_8_/Ni_3_S_2_‖Ni_3_Fe is pretty competitive among recently reported electrocatalysts (Table S1†).

## 4. Conclusions

In summary, we have demonstrated a simple and one-step hydrothermal approach for synthesis of Ni_4.5_Fe_4.5_S_8_/Ni_3_S_2_ on Ni_3_Fe alloy foams. Direct growth of electrocatalyst with mountainous terrain on bulk foam endows this system with felicitous stability in strongly alkaline electrolytes, evidenced by SEM, XRD and XPS characterization after stability tests. The lowest electron transfer resistance shown by EIS further enhances the performance of Ni_4.5_Fe_4.5_S_8_/Ni_3_S_2_. Formation of thin metal metal-hydro (oxide) shell around the electrocatalyst and conductive core (Ni_4.5_Fe_4.5_S_8_/Ni_3_S_2_) inside may work together to boost the overall activity. With these excellent figures of merit, it requires an overpotential of only 264 mV to drive 100 mA cm^−2^ with its catalytic activity being maintained for at least 20 h in 1 M KOH. Further study entails understanding the working mechanism of this system in combination with more characterization methods and theoretical calculation. It is believed that the design strategy developed in this work may provide a new perspective for designing other advanced composite materials catalysts for electrocatalysis application.

## Conflicts of interest

There are no conflicts to declare.

## Supplementary Material

RA-009-C9RA00724E-s001
