# Supplementary material for: Synthesis of Ni4.5Fe4.5S8/Ni3S2 film on Ni3Fe alloy foam as an excellent electrocatalyst for the oxygen evolution reaction
Source: RSC Adv. 2019 Apr 2;9(18):10231–6. doi: 10.1039/c9ra00724e (PMC9062387; doi:10.1039/c9ra00724e)
Supplement: RA-009-C9RA00724E-s001 [file RA-009-C9RA00724E-s001.pdf]

Electronic Supplementary Material (ESI) for RSC Advances.
This journal is © The Royal Society of Chemistry 2019

Synthesis of $Ni_{4.5}Fe_{4.5}S_8/Ni_3S_2$ Film on Ni_3Fe Foam as an Excellent Electrocatalyst for Oxygen Evolution Reaction

Shili Qin,[a] Jinlong Lei,[a] Yun Xiong,[b] Xiaohu Xu,[c] Xinhua Geng*[a] and Jiahai Wang*[a]

[a] Department of Chemistry and Chemical Engineering, Guangzhou Key Laboratory for Environmentally Functional Materials and Technology, Guangzhou University, Guangzhou 510006, P.R. China.

[b] Wuhan Economic and Technological Development Zone, Wuhan HydraV Fuel Cell Tech. Co., Ltd, Wuhan 430056, P.R. China.

[c] Key Laboratory of Spectral Measurement and Analysis of Shanxi Province, Shanxi Normal University, Linfen 041004, P.R. China.

E-mail: ccgxinh1230@gzhu.edu.cn; jiahaiwang@gzhu.edu.cn

Tel: +86-18816801579

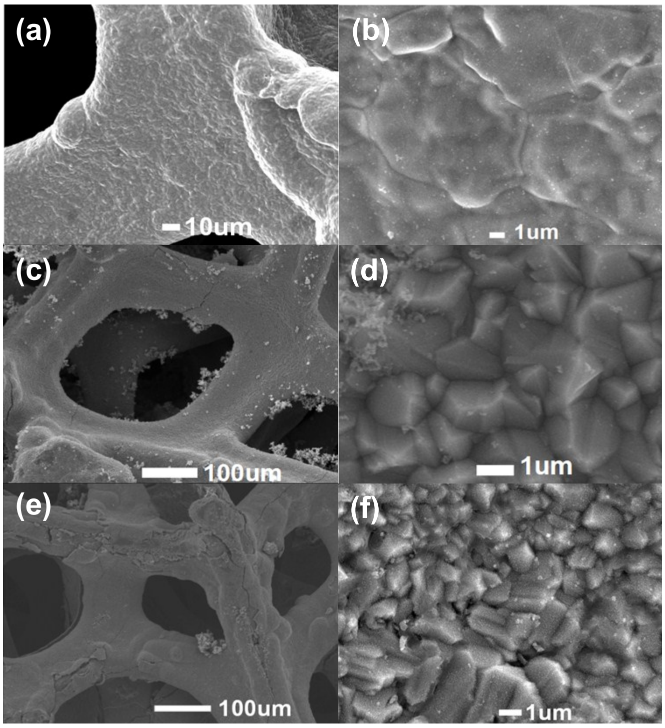

Fig. S1 SEM images of (a, b) Ni_3Fe foam. (c, d) $Ni_{4.5}Fe_{4.5}S_8/Ni_3S_2||Ni_3Fe$ sample. (e, f) $Ni_{4.5}Fe_{4.5}S_8/Ni_3S_2||Ni_3Fe$ sample after 1000 CV cycles.

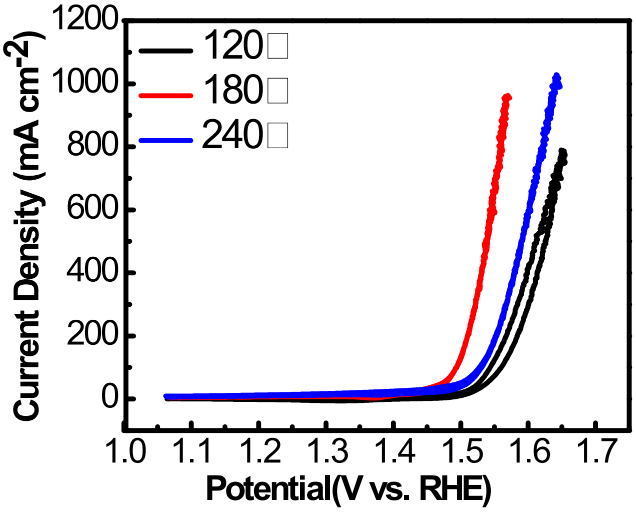

Fig. S2 The OER of $Ni_{4.5}Fe_{4.5}S_8/Ni_3S_2$||$Ni_3Fe$ prepared in different temperatures.

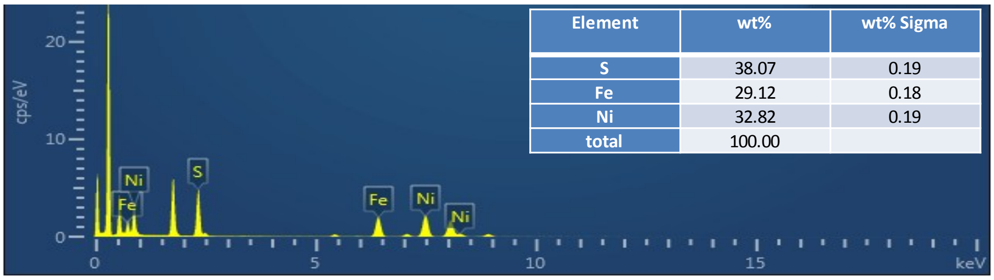

Fig. S3 EDS pattern of $Ni_{4.5}Fe_{4.5}S_8/Ni_3S_2$||$Ni_3Fe$ sample.

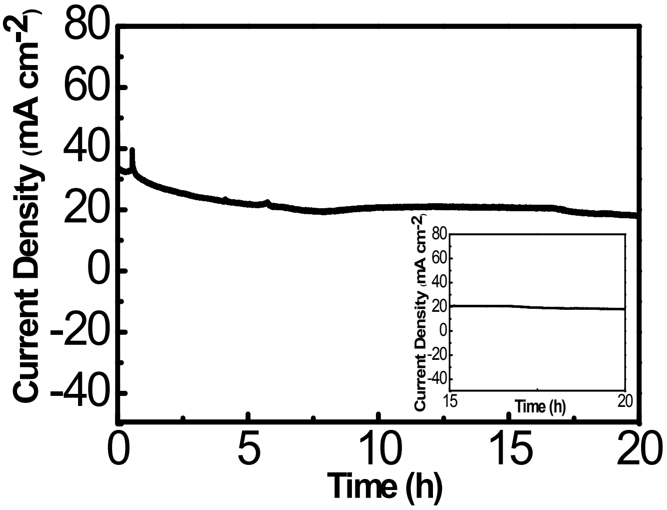

Fig.S4 Chronoamperometric durability test at a constant overpotential of 230 mV.

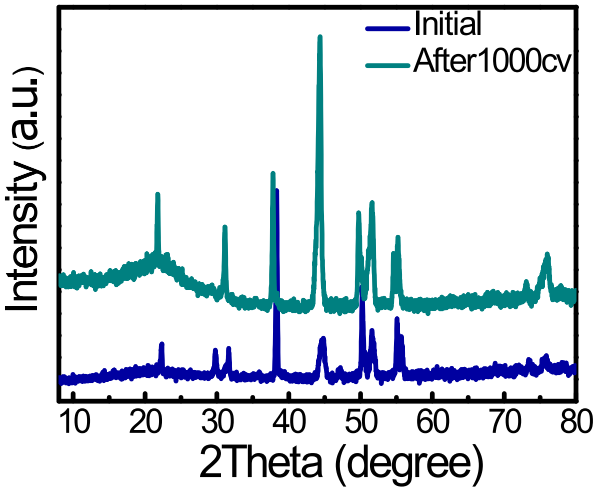

Fig. S5 XRD pattern of $Ni_{4.5}Fe_{4.5}S_8/Ni_3S_2$||$Ni_3Fe$ and after 1000 CV cycles.

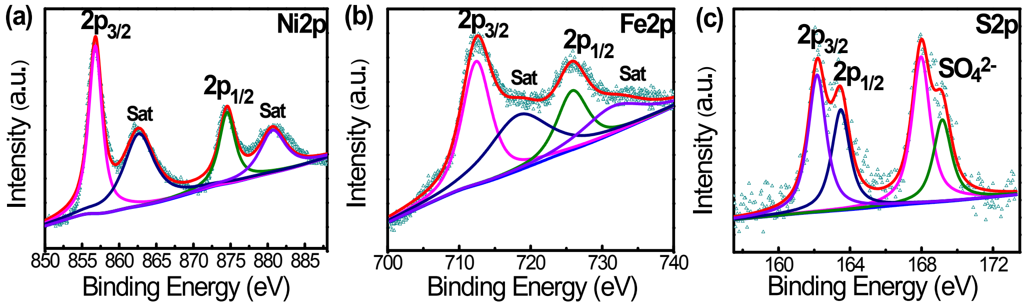

Fig. S6 XPS spectra for (a) Ni2p. (b) Fe2p. (c) S2p of $Ni_{4.5}Fe_{4.5}S_8/Ni_3S_2$||$Ni_3Fe$ after 1000CV.

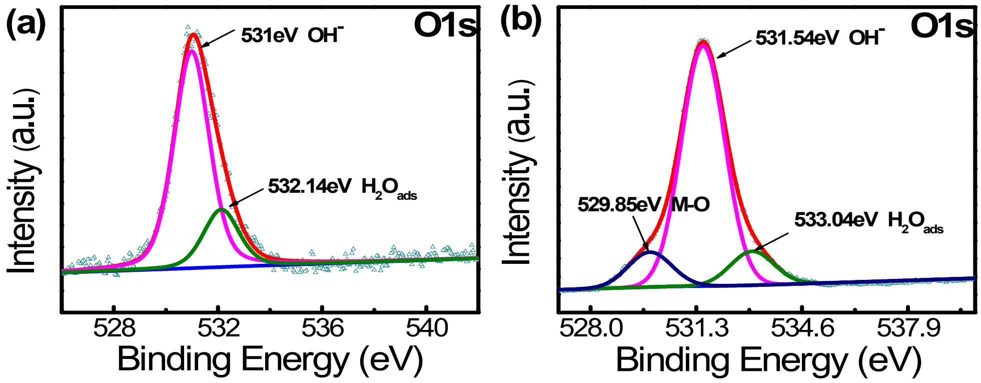

Fig. S7 XPS spectra for O1s of (a) $Ni_{4.5}Fe_{4.5}S_8/Ni_3S_2$||$Ni_3Fe$. (b) After 1000 CV.

Table S1 A comparison of the OER overpotentials for the reported electrocatalysts.

Catalysts	η at 10 mA cm^{-2} (mV)	η at 100 mA cm^{-2} (mV)	Tafel slope (mV dec^{-1})	Substrate	References
Fe- Ni_3S_2	---	253	65.5	NF	J. Mater. Chem. A, 2015.3(46):p.23207-23212.
Fe- Ni_3S_2	282	490	54	NiFe	Small, 2017. 13(18): p. 1604161.
N-Ni_3S_2	---	330	70	NF	Adv. Mater.2017. 29(30).
NiFeS	65	189	119.4	NF	J. Mater. Chem. A, 2016 4(35):p.13499-13508.
NiFeS	---	180	55	NF	J. Mater. Chem. A, 2016.4(42):p.16394-16402.
$MoS_2/Fe_5Ni_4S_8$	204	240	48	NiFe	Adv. Mater, 2018: p. e1803151.
NiFe-NC	271	340	48	---	ACS. Appl. Mater Interfaces.2017.9(48):p.41906-41915
NiFeOF	320	---	38	Stainless steel	ACS Catalysis.2017. 7(12): p. 8406-8412.
Fe-$Ni(OH)_2$	235	---	51.5	NF	Chem. Commun (Camb). 2018. 54(5): p. 463-466.
Ni_3FeN	190	---	72	CC	ACS Appl. Mater Interfaces. 2018. 10(4): p. 3699-3706.
$(NiFe)S_2$	260	410	56.2	---	Chem. Sci. 2018. 9(10): p. 2762-2767.
NiFe	283	---	53	NiFe	ACS Appl. Mater Interfaces. 2017. 9(34): p.28627-28634
$NixCo_3{-}xS_4/Ni_3S_2$	---	300	95	NF	Nano Energy. 2017. 35: p. 161-170.
Co-$NiO/NiFe_2O_4$	186	220	35	NF	J. Mater. Chem. A, 2018. 6(1): p. 167-178.
Ni−Fe−P	271	---	53	---	ACS. Appl. Mater Interfaces.2017. 9(31): p. 26134-26142.
Ni_2P@NiFe-LDH	205	230	32	NF	Chem. Sci.2018. 9(5): p.1375-1384.
$Ni_{4.5}Fe_{4.5}S_8/Ni_3S_2$	166	264	63.15	Ni_3Fe	This work
